# Raman imaging at biological interfaces: applications in breast cancer diagnosis

**DOI:** 10.1186/1476-4598-12-48

**Published:** 2013-05-24

**Authors:** Jakub Surmacki, Jacek Musial, Radzislaw Kordek, Halina Abramczyk

**Affiliations:** 1 Laboratory of Laser Molecular Spectroscopy, Institute of Applied Radiation Chemistry, Lodz University of Technology, Wroblewskiego 15, Lodz 93-590, Poland; 2 Department of Pathology, Chair of Oncology, Medical University of Lodz, Paderewskiego 4, Lodz 93-509, Poland

**Keywords:** Breast cancer biochemical imaging, Interfacial water, Raman imaging, IR spectroscopy

## Abstract

**Background:**

One of the most important areas of Raman medical diagnostics is identification and characterization of cancerous and noncancerous tissues. The methods based on Raman scattering has shown significant potential for probing human breast tissue to provide valuable information for early diagnosis of breast cancer. A vibrational fingerprint from the biological tissue provides information which can be used to identify, characterize and discriminate structures in breast tissue, both in the normal and cancerous environment.

**Results:**

The paper reviews recent progress in understanding structure and interactions at biological interfaces of the human tissue by using confocal Raman imaging and IR spectroscopy. The important differences between the noncancerous and cancerous human breast tissues were found in regions characteristic for vibrations of carotenoids, fatty acids, proteins, and interfacial water. Particular attention was paid to the role played by unsaturated fatty acids and their derivatives as well as carotenoids and interfacial water.

**Conclusions:**

We demonstrate that Raman imaging has reached a clinically relevant level in regard to breast cancer diagnosis applications. The results presented in the paper may have serious implications on understanding mechanisms of interactions in living cells under realistically crowded conditions of biological tissue.

## Background

The most important factors in improving the survival rate for cancer patients are: reliable diagnosis for early detection, early treatment, and treatment response monitoring providing with unequivocal data. There is a profound clinical need for diagnostic tools to achieve these goals.

The current diagnosis or prevention methods are based on X-Ray mammography, magnetic resonance imaging (MRI), positron emission tomography (PET) and ultrasonography. X-Ray mammography is the golden standard of imaging examination used for breast cancer diagnosis. However, there are well-known limitations in terms of sensitivity and specificity, especially when scanning patients with high breast density, common in younger women. This leads to relatively unsatisfying levels of false positive and false negative results, as high as 75% and 34%, respectively [1-3]. Moreover, mammography is not a specific screening method, as it does not allow to differentiate between benign and malignant tumors. The second standard method – ultrasonography is a fast and low-cost technique, but has a low spatial resolution [4]. The MRI technique offers a good contrast between different soft tissues of the body, but has many drawbacks, like the long duration of the examination and the cost of the instrument. It also causes side effects in the patients like hyperthermia or a negative impact on the patient’s kidneys, related to the use of gadolinium contrast agent [5]. Positron Emission Tomography (PET) is an appealing complementary technique for breast imaging, but the image resolution of PET is low and typically ranges between 4 and 7 mm. To improve resolution and contrast between normal and cancerous breast tissue a dedicated breast PET – Positron Emission Mammography (PEM) has been developed [6-8].

To overcome major drawbacks of the standard techniques, the optical methods such as diffuse optical tomography–fluorescence mediated tomography [9,10], photo-acoustic imaging [11], acousto-optical imaging [12] have been developed to obtain a more accurate, rapid, inexpensive, and non-destructive method of imaging. The optical imaging systems are ideally suited for early detection of intraepithelial diseases, including most cancers, and allow assessing tumor margins and response to therapy. Optical methods offer several significant advantages over the routine clinical imaging methods, including: a) non-invasiveness through the use of safe, non-ionizing radiation, b) display of contrast between soft tissues based on optical properties of the tissue, c) facility for continuous bedside monitoring, d) high spatial resolution (less than 1 micron lateral resolution in visible range). Almost all of the widely used methods of molecular diagnostics centered on protein separation, amplification, and gene expression in target proteins and nucleic acids involve fluorescence spectroscopy. The confocal, laser-based fluorescence microscopy became a golden standard for optical imaging and molecular diagnostics [13]. The disadvantage of fluorescence is that the multiplexing capability of this technique is limited due to the broad emission profile of fluorophores. This results in difficulty in deconvoluting mixtures of signals.

Raman spectroscopy has many advantages over fluorescence [14]. First, Raman spectroscopy needs no external labeling as the contrast is based on endogeneous optical properties of the biological tissue. The contrast is generated in the cross section for Raman scattering due to induced polarizability generated in the tissue upon irradiation. Raman scattering is inelastic scattering, and measuring the difference between the energy of the incident photons and scattered photons one can obtain the information about vibrations. Therefore, biochemical signatures of the molecules obtained from the Raman spectroscopy are richer than those from the fluorescence spectroscopy. For example–nucleic acids, lipids, biological chromophores and proteins are characterized by narrow vibrational peaks in different spectral regions, in contrast to broad, largely unspecific emission profiles obtained in the fluorescence method. Second, Raman imaging (RI) has now reached a level of sophistication that makes it competitive with more conventional methods of confocal fluorescence microscopy in terms of sensitivity, specificity, and spatial resolution.

Optical Raman imaging has emerged as a new modality which enables real time, non-invasive, high resolution imaging of epithelial tissue, with a particular focus in this paper on breast cancer diagnosis [15-34]. The method has a potential to replace the conventional biopsy and histopathological analysis by an optical Raman biopsy [35-39]. Due to confocal microendoscopy advancements, optically fiber coupled spectrometers and semiconductor technology it becomes possible to perform the in vivo mode of operation in real time.

Raman scattering efficiency can be enhanced by factors >10^8^ when a substance is adsorbed on or near rough metal surfaces. The Raman scattering amplification associated with this phenomenon is known as surface-enhanced Raman scattering (SERS) spectroscopy [40]. These huge increases in Raman scattering are primarily caused by the increased intensity of the electromagnetic fields present at the surface of these metals. SERS preserves the essential features of Raman scattering, yet the surface enhancement provided by the metal nanoparticle allows unique spectra to be acquired from a variety of adsorbed species.

The enhancement can further increase even up to 11 orders and reach a limit of detection of 10^-16^ M in surface-enhanced resonance Raman spectroscopy (SERRS) [41,42], 13 orders in surface-enhanced Raman scattering–scanning near-field optical microscopy (SERS–SNOM) [31] or even 14–15 orders of magnitude and detection down to 10^-10^–10^-14^ M in non-linear optical microscopy (e.g. coherent anti-Stokes Raman scattering (CARS)) [43-46].

Therefore, with further advancements of SERS combined with highly sensitive SERS active probes, Raman techniques may open up a new direction in nanomedicine, and bioimaging for high-sensitive high-throughput signal detection, even down to the single-molecule level [44-46].

There is increasing evidence that Raman imaging can be utilized to understand molecular mechanisms of breast cancer [21,47,48]. It also has potential to be employed in clinical applications for early diagnosis, recurrence prediction and response to treatment measurement. Properly chosen optically active contrast agents and targeted biomarkers that monitor functional features of cancer can greatly contribute to the development of nucleic acid and protein-based diagnostic tests by Raman methods. The design of proper SERS–based biotags requires a complex interplay of biological interactions for increasing the number of fluorescent proteins or nanoparticles, which is currently at early stages of research and development [48,49].

This paper will focus on new developments in breast cancer biochemical imaging, as well as exploring the potential of clinical applications. The aim of the present study is to demonstrate that the label-free Raman imaging has the ability to accurately characterize breast cancer tissue and distinguish between noncancerous and cancerous types. We will demonstrate how this knowledge contributes to new branches of diagnostic developments for breast cancer diseases. The results presented in this paper demonstrate that Raman imaging has significant potential for probing human breast tissue to provide complementary data in the early diagnosis of breast cancer.

## Results

In this section we present the results of the Raman and IR studies on the noncancerous and cancerous human breast tissues. Having reached this point when we have established the proper protocol of tissue preparation to obtain reliable results from IR and Raman measurements, we will explore vibrational spectroscopy and imaging for a variety of biomedical applications, particularly molecular diagnostics of breast cancer. Details of the protocol are provided in Additional file 1, available at [URL].

**Figure 1:**
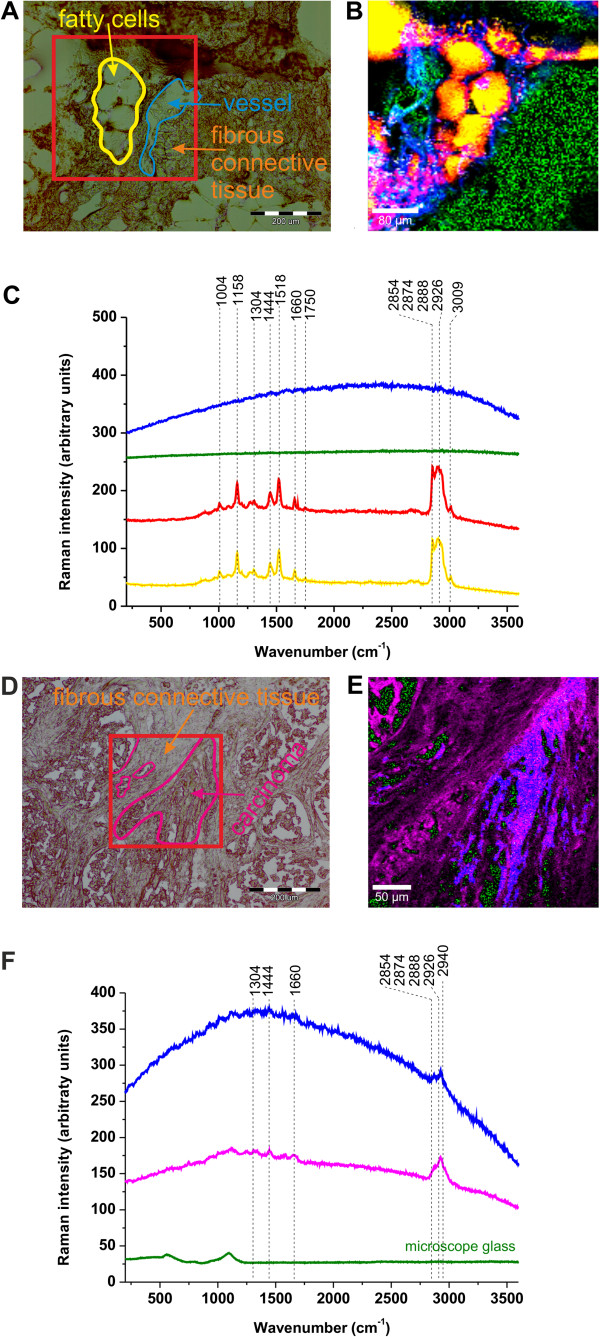
**Raman image and spectra of the noncancerous and cancerous breast tissue of the patient P81.** Noncancerous breast tissue: (**A**) Microscope image, (**B**) Raman image (400x400 μm) from the region marked in (**A**), (**C**) Raman spectra (integration time: 0.05 s). Cancerous breast tissue: (**D**) Microscope image, (**E**) Raman image (300x300 μm) from the region marked in (**D**), (**F**) Raman spectra (integration time: 0.036 s). The colors of the spectra correspond to the colors in the image. Mixed areas are displayed as mixed colors.

Figure 1(A,B,C) shows the microscope image, a typical Raman image, and Raman spectra of the noncancerous human breast tissue surrounding the tumor from the safety margin for one of the patients. The total number of patients used in Raman measurements is 200 [15-21]. One can see that the Raman images reveal distinct structures corresponding to inhomogeneous distribution of different compounds in the monitored area. At the first glance, the Raman image (Figure 1(B)) spectacularly resembles a microscope image (Figure 1(A)). The advantage of the ‘Raman biopsy’ is that it provides direct biochemical information (vibrational fingerprint) in real time and is not prone to subjective interpretations. Moreover, it monitors the biological tissue structures without any external agents, in contrast to histological assessment [18-21].

In order to identify human breast structures and the corresponding biochemical constituents that produce the Raman vibrational pattern of behavior, we have recorded the Raman image for the cancerous breast tissue of the same patient P81. Figure 1(D,E,F) shows the typical microscope image, a Raman image and Raman spectra of the breast cancerous tissue (infiltrating ductal carcinoma) (of the same patient as in Figure 1(A,B,C). These Raman spectra have been used as the references basis spectra (see Additional file 1 [URL]) to produce the Raman images.

The first observation that one makes when confronting the results obtained are noticeable differences between the Raman spectra and Raman images of the noncancerous breast tissue surrounding tumor from the safety margin (Figure 1(B,C)) and the cancerous breast tissue from the tumor mass (Figure 1(E,F)). The yellow areas in Figure 1(B) represent adipose tissue with red areas corresponding to the membranes of the adipose cells filling spaces around the lobules and the ducts. In contrast, the blue and green colors represent strongly autofluorescent structures. The pink and red areas shown in Figure 1(E) represent proteins in the cancerous breast tissue, which are not visible in the normal tissue in Figure 1(B). This finding corresponds to the fact that in contrast to normal cells, the abnormal cells divide in uncontrolled process of cell growth that synthesizes large amounts of proteins.

Significant differences observed in Raman spectra indicate marked distinctions in biochemical composition and distribution of biochemical components. In the next section we will demonstrate that the contrast in Raman images is dominated by carotenoids, fatty acids, proteins and water. We will show that the normal breast tissue surrounding the tumor contains a markedly higher concentration of fatty acids and carotenoids as compared to that of the cancerous tissue. The higher β-carotene content in normal breast tissue was also found by diffuse reflectance spectroscopy [50]. The cancerous breast tissue has a different profile of vibrational features indicating distinct lipids composition and a much higher contribution from proteins.

## Discussion

**Figure 2:**
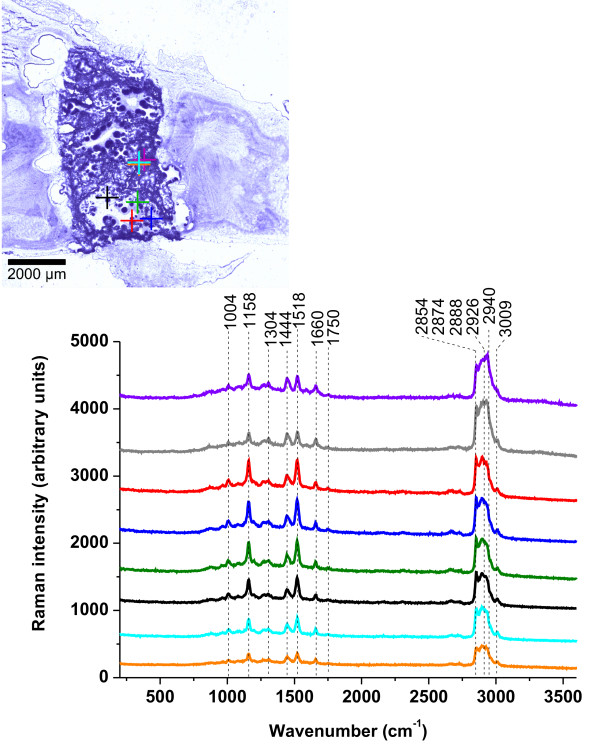
**Raman spectra of the noncancerous tissue of the patient P81.** Raman spectra were obtained from various points in the sample from the regions marked with crosses. The colors of the spectra correspond to the colors of the crosses.

In the view of the results presented in the previous section we first need to identify the main biochemical constituents of human breast tissue that contribute to the Raman vibrational pattern of behaviour. Figure 2 shows the typical Raman spectra of the noncancerous tissue, which were obtained from various points of the sample.

The Raman spectra of the noncancerous tissue shows peaks at 877, 1004, 1158, 1304, 1444, 1518, 1660, 1750, 2854, 2888, 2926, and 3009 cm^-1^. The Raman spectra at 1158 and 1518 cm^-1^ have been assigned to carotenoids. The bands at 1158 and 1518 cm^-1^ represent the stretching mode of C-C and C=C bond of the polyene chain in carotenoids [20,21]. The Raman peaks at 1444, 1660, 1750, 2854, 2888, 2926 and 3009 cm^-1^ have been assigned to fatty acids of the adipose tissue contained in the normal breast structure. The comparison with the Raman spectra of the essential fatty acids ω-3 and ω-6 shows that the normal tissue is dominated primarily by oleic acid derivatives [19,21]. The assignment of the Raman peaks observed in the noncancerous breast tissue is presented in Additional file 1: Table S1 [URL].

**Figure 3:**
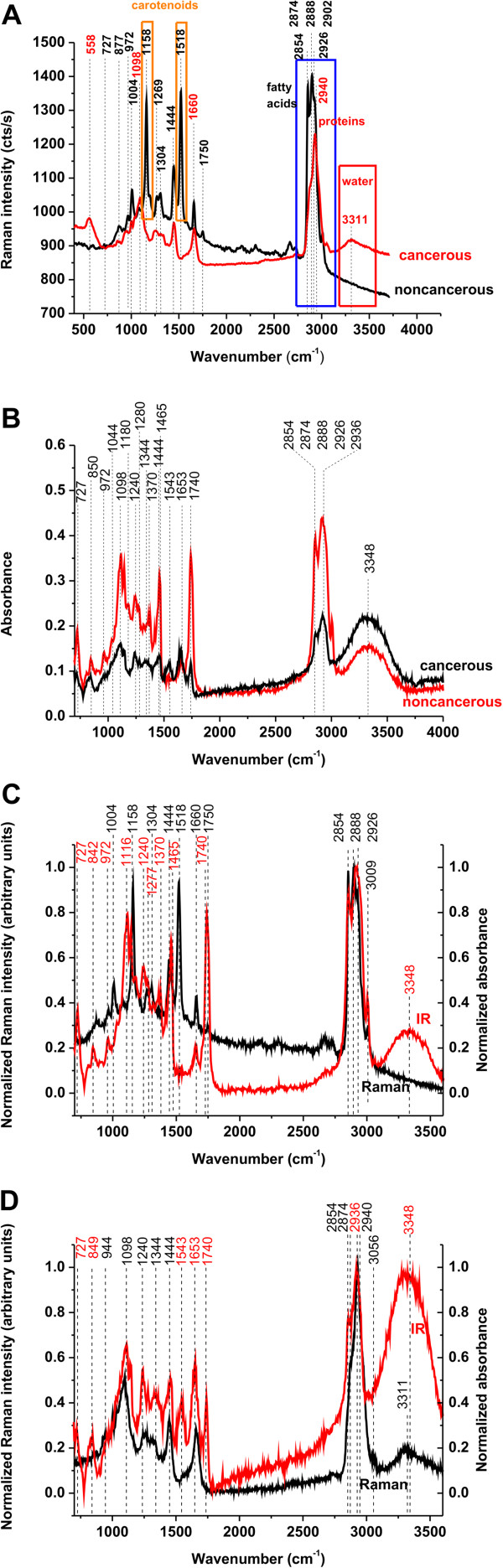
**Raman and IR spectra for the noncancerous and cancerous breast tissues (infiltrating ductal carcinoma).** (**A**) Raman spectra of the patient P81; (**B**) IR spectra of the patient P83; (**C**) Raman spectrum for the noncancerous normal breast tissues of the patient P81 and IR spectrum for the noncancerous normal breast tissues of the patient P83; (**D**) Raman spectrum for the cancerous breast tissues of the patient P81 and IR spectrum for the cancerous breast tissues of the patient P83.

Figure 3(A) compares the Raman spectra for the noncancerous and cancerous breast tissues of the patient P81 (infiltrating ductal carcinoma). The cancerous tissue displays only a limited number of Raman peaks at 558, 1098, 1269, 1444, 1660, 2888, 2926, 2940 and 3311cm^-1^. The assignment of the Raman peaks observed in the cancerous breast tissue is presented in Additional file 1: Table S2 [URL].

One of the most remarkable aspects of the comparison are marked differences between the Raman spectra of the noncancerous and cancerous tissues. The most pronounced differences can be seen in the biochemical composition and distribution of carotenoids (1158 and 1518 cm^-1^), lipids (2800–3000 cm^-1^) and water represented by the OH stretching band at 3311 cm^-1^.

To obtain complementary information on vibrational features of the normal and cancerous tissues, it would be very interesting to compare the Raman spectra with IR spectra. Figure 3(B) shows the comparison between the IR spectra of the noncancerous tissue and cancerous breast tissue.

The comparison between the IR results of the patient P83 (infiltrating ductal carcinoma) presented in Figure 3(B) demonstrates that the vibrational peaks for the noncancerous and cancerous breast tissues are very similar. The differences in Figure 3(B) are related to the magnitudes of absorbance in the region of 2800–3500 cm^-1^, 1740 cm^-1^, 1444 cm^-1^, 972 cm^-1^.

In the view of the results presented in Figure 3(A) and (B) it is clearly visible that the Raman method demonstrates a markedly higher specificity allowing to distinguishing between the normal and cancerous breast tissues, thus having the potential to be a better diagnostic tool in breast cancer pathology.

In order to rationalize the vibrational features of the cancer pathology monitored by IR and Raman spectroscopy we have compared the results for the noncancerous and cancerous breast tissues. Figure 3(C) compares Raman and IR spectra for the noncancerous breast tissues.

A detailed inspection into Figure 3(C) demonstrates that the vibrations originating from carotenoids at 1158 cm^-1^ and 1518 cm^-1^, which are the strongest signals in the Raman spectrum, are not visible in the IR spectrum. The reason is quite obvious, and that is because the excitation with 514 nm leads to the resonance Raman enhancement of carotenoids, which is not present in IR measurements. The IR spectra of the noncancerous tissue shows peaks at 1444, 1660, 2854, 2888, 2926 cm^-1^ just like Raman spectra. Additional peaks at 842, 972, 1116, 1240, 1560, 1740 cm^-1^ are clearly visible in the IR spectrum. The marked distinctions can be observed in the lipid-protein profiles in the region of 2800–3000 cm^-1^, where the contribution from the monounsaturated fatty acids that are common constituents of triglycerides of the adipose tissue dominates the Raman spectrum [19] in contrast to the IR spectra that demonstrate a more protein like profile. These differences originate from different selection rules for the Raman spectroscopy and IR spectroscopy. Namely, a change of molecular polarization corresponding to deformation of the electronic cloud around the chemical bound with respect to the vibrational coordinate is required for a molecule to exhibit a Raman signal. In contrast, a change of molecular dipole with respect to the vibrational coordinate is required for a molecule to exhibit an IR signal.

The most pronounced differences can be seen in the region of water represented by the OH stretching vibrations, a strong IR band at 3348 cm^-1^ is observed as opposed to the Raman spectrum, where no water signal is present.

**Figure 4:**
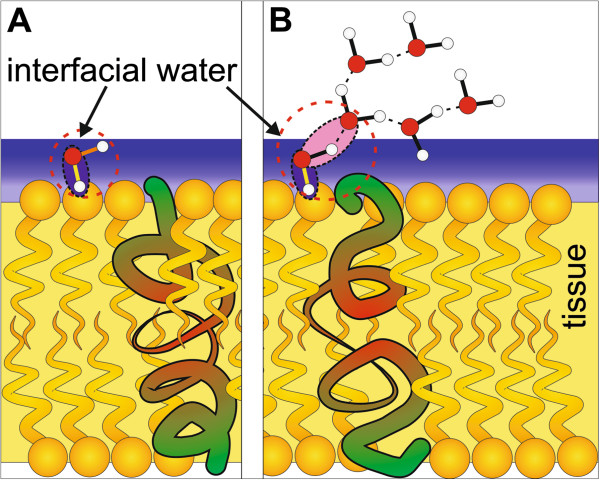
**H**-**bond interactions of interfacial water at biological interfaces.** (**A**) dry sample, (**B**) wet sample; the blue line through the cartoon represents the tissue/water interfaces. The orange and green sites represent the hydrophilic moieties on the surface of the tissue. The red and white circles represent the oxygen and hydrogen atoms in water molecules, respectively.

Figure 3(D) compares Raman and IR spectra for the cancerous breast tissues. In contrast to the noncancerous tissue the differences are less spectacular. First, the Raman spectra of the cancerous tissue show no signals from carotenoids like IR spectra. Second, the lipid-protein profiles of IR and Raman spectra in the region 2800–3000 cm^-1^ are similar, unlike in the case of the normal tissue. Third, both Raman and IR spectra demonstrate contribution from water. The apparently single bands are observed at 3348 cm^-1^ for IR and 3311 cm^-1^ for Raman, which we have assigned to the OH stretching mode of water. The main difficulty in interpretation of the Raman (and IR bands) in the region of 3000–3600 cm^-1^ comes from the fact that location of bands of water may overlap with the N-H stretching bands of proteins (amide A at 3365 cm^-1^) and N-H stretching vibrations of DNA. The detailed discussion of this assignment has been presented in reference [24]. It is worth emphasizing that the OH stretching region of water in the breast tissue with the single band differs markedly from that of the bulk water represented by a double structure with the maximum band positions at 3258 cm^-1^ and 3410 cm^-1^[18]. It indicates that the characteristic vibrational features of water molecules near a biological interface, where the H-bond network gets locally disrupted, differ significantly from those of bulk water due to interactions with the hydrophilic sites of the tissue. Water in the breast tissue is very likely present as interfacial molecules, with one bond hydrogen bonded to some hydrophilic moieties in the tissue (e.g. proteins or lipid head), and the second bond being free (Figure 4(A)) or involved in H-bond interactions with another water molecules (Figure 4(B)) [18]. The single Raman peak at 3311 cm^-1^ in Figure 3(A) (3348 cm^-1^ in IR, Figure 3(B)) has been assigned to the stretching vibration of the OH bond, which is decoupled from the other OH bond of a water molecule. The mechanism of decoupling is related to breaking of the C_2ν_ symmetry similar to that observed in water diluted in deuterated water, where the HDO species are generated as a result of isotopic exchange. Breaking of the C_2v_ symmetry comes from nonequivalent interactions of the O-H bonds of the water molecules in the biological tissue - one of the OH bonds interacts with the hydrophilic sites of the tissue (blue OH bond in Figure 4), the other OH is almost quasi free and not involved in any H-bond interactions (dry sample in Figure 4(A)–when there is a limited number of water molecules at the interface) or interacts with other water molecules of the H-bond network (wet sample in Figure 4(B))–when there is a sufficient number of water molecules at the interface). This mechanism illustrated in Figure 4 seems to occur for the surface water at biological interfaces.

The important question arises why IR spectroscopy identifies interfacial water in the spectral region of 3000–3700 cm^-1^ both in the noncancerous and in the cancerous tissues (see Figure 3(B) and Additional file 1: Figure S3, [URL]) in contrast to Raman spectroscopy, where only cancerous tissue exhibits vibrational features of water (see Figure 3(A)). It can be explained by the different configurations of the optical paths in Raman and IR techniques (Additional file 1: Figure S1, [URL]). The Raman spectroscopy measures the Raman signal scattered back from the tissue. The IR methods measures the signal transmitted through the tissue thin section attached to the window (BaF_2_). Therefore, the layer of external, ambient water is confined between the tissue section and the surface of the window, giving the contribution to the IR spectrum both in the noncancerous and the cancerous tissues. The ambient water cannot be removed by the reference beam, because the amount of water attached to the empty window differs from that confined under the tissue slice. In the Raman measurements the laser penetration depth is around 30 μm at 514 nm and allows to reaching the layer of water between the microscope glass and the tissue slice of 2 μm. However, in the confocal Raman microscopy the laser beam is focused exactly on the tissue and the back scattered Raman signal originates only from the tissue. As a result, the Raman signal is dominated by the water molecules confined within the tissue in contrast to the IR signal originating largely from the absorption of water from the space between the tissue and the window (Additional file 1: Figure S1, [URL]).

The detailed inspection into Figure 3(A) shows the spectacular differences in the Raman spectra of the OH band of water in the noncancerous and cancerous breast tissues. The noncancerous tissue shows a negligible signal of water when compared to the cancerous tissue where the signal peak at 3311 cm^-1^ is observed. The lack of water is likely due to higher content of hydrophobic adipose tissue in the noncancerous tissue. Indeed, the Raman images presented in this paper show evidently that the breast structure of the noncancerous tissue contains a significantly higher content of the adipose cells (demonstrated in the Raman imaging by the yellow color in Figure 1(B) than the cancerous tissue (Figure 1(E)) that shows negligible amount of the adipose tissue.

In the view of the results presented so far one can state that there are four important Raman markers of breast tissue - carotenoids, fatty acids, proteins and water. Our results demonstrate that there is an intriguing correlation between the amount of monounsaturated fatty acids, carotenoids and water in the breast tissue. First, we will concentrate on the link between carotenoids and monounsaturated fatty acids. Recently, we have shown that oleic acid exists as a separate phase within the noncancerous human breast tissue [19]. The question arises whether we are capable of learning from Raman imaging about areas of the breast tissue structure in which carotenoids and monounsaturated fatty acids are accumulated. Answering this question may be related to the primary mechanisms of carcinogenesis. Several physiological functions have been attributed to carotenoids, particularly an upregulation of gap junctional communication (GJC) in connexins [51,52]. There is growing evidence that the GJC plays an important role in the regulation of morphogenesis, cell differentiation, secretion of hormones, and growth as well as in human diseases including human breast cancer [53].

**Figure 5:**
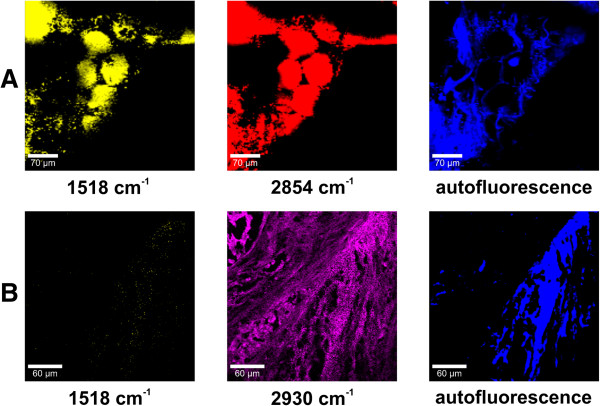
**Raman images.** (**A**) noncancerous and (**B**) cancerous breast tissue, Filters: carotenoids (1518 cm^-1^), monounsaturated fatty acids (2854 cm^-1^), proteins (2930 cm^-1^) and autofluorescence (1800 cm^-1^).

In order to monitor accumulation of carotenoids and fatty acids in localized regions of the tissue presented in Figures 1(B) and (E) we have employed various spectral filters in the Raman images to analyze different areas of the noncancerous and cancerous breast tissues. Figure 5 shows the Raman images for the noncancerous (Figure 5(A)) and cancerous (Figure 5(B)) breast tissue for the filters at 1518 cm^-1^, 2854 cm^-1^, 2930 cm^-1^ and 1800 cm^-1^ corresponding to the vibrational frequency of carotenoids, oleic acid, proteins and autofluorescence, respectively. One can see that for the noncancerous tissue the Raman image at the filter 1518 cm^-1^ (Figure 5(A)) illustrating the distributions of carotenoids is almost identical as that for the filter 2854 cm^-1^ for unsaturated fatty acids and triglycerides. It clearly indicates that the unsaturated fatty acids and triglycerides of the adipose tissue act as a dynamic reservoir that supplies carotenoids to the human organs. In contrast, the cancerous breast tissue does not contain any carotenoids as demonstrated by Figure 5(B) at the filter 1518 cm^-1^. The pink areas in Figure 5(B) at the filter 2930 cm^-1^ illustrate the distribution of proteins in the cancerous breast tissue. The comparison of the images of fluorophores in the tissues (at the filter 1800 cm^-1^) in Figure 5(A) and (B) demonstrates that the fluorescent species (blue areas) in the cancerous tissue are accumulated in similar regions as the proteins (pink areas) in contrast to the noncancerous tissue, where the fluorophores are accumulated in the regions complementary to those of the monounsaturated fatty acids and carotenoids (yellow and red areas).

**Figure 6:**
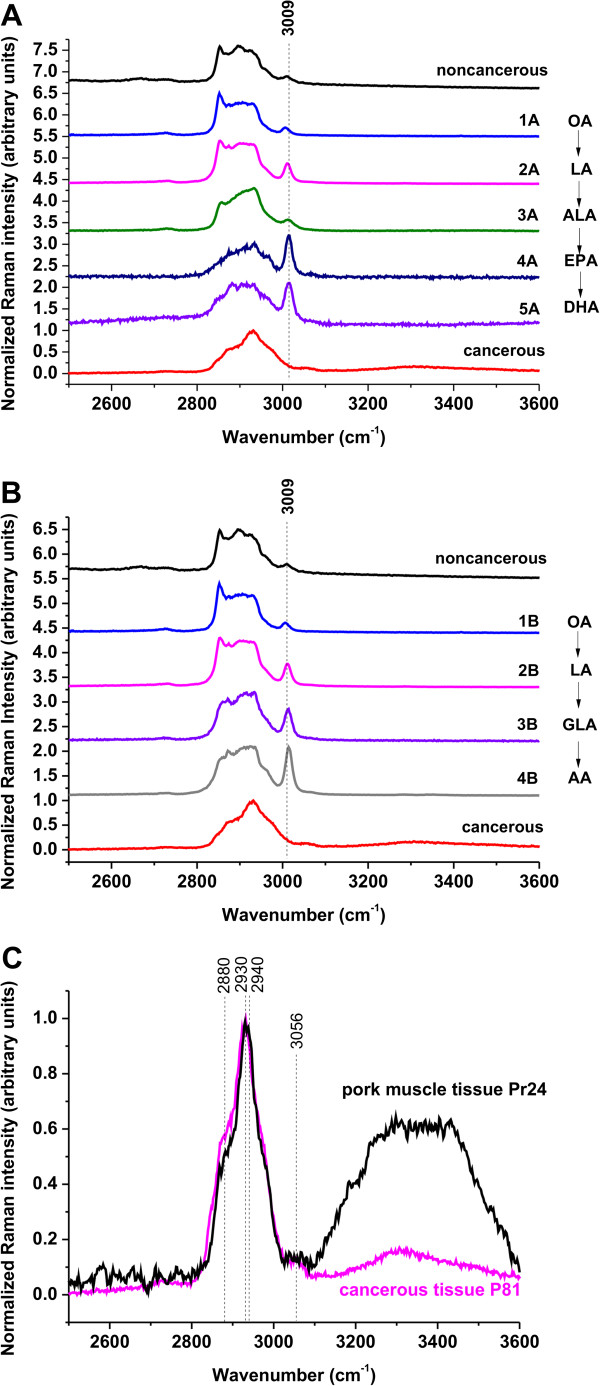
**Raman spectra of fatty acids compared with the Raman spectra of cancerous and noncancerous breast tissues.** (**A**) 1A - oleic acid (OA), 2A - linoleic acid (LA), 3A - α-linolenic acid (ALA), 4A - eicosapentaenoic acid (EPA), 5A - docosahexaenoic acid (DHA), (**B**) 1B - oleic acid (OA), 2B - linoleic acid (LA), 3B - γ-linolenic acid (GLA), 4B - arachidonic acid (AA). (**C**) Comparison between the Raman spectrum of the cancerous tissue (patient P81) and the spectrum of a muscle tissue from pork meat (Pr 24).

The main difficulty in interpretation of the Raman and IR bands in the region of 2800–3000 cm^-1^ comes from the fact that the spectral region of the bands of fatty acids and lipids overlap with the bands of proteins. The main protein band near 2940 cm^-1^ attributed to aromatic and aliphatic amino acids and many other amino acids overlaps with the characteristic C-H bands of fatty acids [24] attributed to a variety of CH, CH_2_, and CH_3_ groups in the side chains. Fortunately, a band at 3009 cm^-1^, corresponding to H-C=C vibrations, which is clearly visible in fatty acids (Figure 6(A,B)), is relatively weak in most proteins and may help to identify dominating components of the tissue in this spectral region.

Figure 6(A,B) compares Raman spectra of selected fatty acids with the Raman spectra of the noncancerous and cancerous breast tissue. Detailed inspection into Figure 6 (A) and (B) shows that the noncancerous tissue contains the 3009 cm^-1^ vibration typical for fatty acids in contrast to the cancerous tissue that reveals no contribution from 3009 cm^-1^ component. To check the contribution from proteins we have compared the Raman spectrum of the cancerous tissue with the spectrum of a muscle tissue taken from pork meat, which is dominated by a protein component. Figure 6(C) shows the comparison of the Raman spectrum of the cancerous breast tissue of the same patient as in the previous figures with the spectrum of a muscle tissue from pork meat. One can see from Figure 6(B) that the profile of the cancerous tissue is very similar to the protein component with significant contribution of the bands at 2930–2940 cm^-1^ from proteins and no sign of contribution of the band at 3009 cm^-1^ from fatty acids. The main band of proteins typically at 2939–2944 cm^-1^ is due to the aromatic and aliphatic amino acids, the charged amino acids and proline, threonine, and histidine. In contrast to the bands near 2930–2940 cm^-1^, the bands near 2880 and 3056 cm^-1^ may reflect predominantly aliphatic and aromatic groups, respectively [54]. The growing contribution from the protein components is a well known indicator of carcinogenesis [55]. Approximately 30% of breast cancers have an amplification of the HER2/neu gene or overexpression of its protein HER2 product.

## Conclusions

This study has illustrated important aspects of Raman reporters that may be used to characterize human breast tissue at a biochemical molecular level. A number of vibrational features in the C-H stretch region change during the process of transformation of the normal breast tissue into the cancerous tissue. These changes may be used to monitor conformational changes of fatty acids, hydrophobic interactions, denaturation, as well as conformational changes of proteins. The biochemical information obtained from the Raman imaging will be particularly useful in monitoring how breast epithelial cells respond to signals from the extracellular matrix (ECM) and how cells lose their normal interactions with the ECM during cancer progression. Raman imaging will help to monitor an increased deposition of ECM proteins and fibroblasts in the stroma surrounding the epithelial cells which is a leading risk factor for breast carcinoma. As a result Raman imaging may help to understand the mechanisms by which stromal density could promote breast carcinoma.

We have found that Raman spectra and Raman images are sensitive indicators of the distribution of main constituents of breast tissue structure such as lipids, proteins, fatty acids, carotenoids and water. The Raman images show evidently that the noncancerous breast tissue contains a significant contribution from triglycerides originating from the adipose tissue filling the spaces around the lobules and ducts, and fatty acids that make up the cell membrane and nuclear membrane. The cancerous tissue is dominated by the protein component. We have found that the water amount confined in the cancerous tissue is markedly higher than in the noncancerous tissue. The OH stretching vibrations of water, C-C,C=C stretching vibrations of carotenoids, 2930–2940 cm^-1^ vibrations of proteins, and 2800–3009 cm^-1^ vibrations of fatty acids can be useful as potential Raman biomarkers to distinguish between the cancerous and the noncancerous human breast tissues. Our results provide experimental evidence for the role played by the lipid-protein-carotenoid profile and cell hydration as factors of particular significance in differentiation of the non-cancerous and cancerous breast tissues.

Our further studies will concentrate on a detailed understanding of interfacial water at the biological surfaces of the cancerous human tissue, where the crowded environment of many biomolecules introduces its own “hydration fingerprint”. It is one of the most important topics in molecular biology because it will not only improve our current knowledge about water itself but also lead to significant advances in our understanding of the structure and process with which confined water is associated (protein folding, hydrophobic assemblies, biological membranes).

## Methods

All procedures were conducted under a protocol approved by the institutional Bioethical Committee at the Medical University of Lodz, Poland (RNN/29/11/KE, RNN/30/11/KE, RNN/31/11/KE).

Detailed methodology is available in Additional file 1, available at [URL].

## Abbreviations

IR: infrared; MRI: magnetic resonance imaging; PET: positron emission tomography; PEM: positron emission mammography; RI: Raman imaging; SERS: surface-enhanced Raman scattering; Microscopy (SERS–SNOM): surface-enhanced Raman scattering–scanning near-field optical; CARS: coherent anti-Stokes Raman scattering; DNA: deoxyribonucleic acid; GJC: gap junctional communication; HER2/neu: human epidermal growth factor receptor 2; OA: Oleic acid; LA: Linoleic acid; ALA: α-linolenic acid; EPA: Eicosapentaenoic acid; DHA: Docosahexaenoic acid; OA: Oleic acid; LA: Linoleic acid; GLA: γ-linolenic acid; AA: Arachidonic acid.

## Competing interests

All authors declare that they do not have any competing interests.

## Authors’ contributions

JS performed most of the experiments, analyzed the data, preparing the figures and tables, JM prepared histological samples, examined histological specimens, RK participated in designed carcinogenesis study, HA participated in designed carcinogenesis study, analyzed the data and interpretation of the results. All authors read and approved the final manuscript.

## Supplementary Material

Additional file 1: Figure S1 Illustration of measurement techniques: transmission IR spectroscopy and confocal Raman scattering microscopy. **Figure S2**. IR spectra of the noncancerous and cancerous (infiltrating ductal carcinoma) human breast tissue slices (patient P81) (**a**) on the microscope glass of paraffin-embeddeded, nonstained tissue ; (**b**) the histological samples after deparaffinization, staining and coating with a standard adhesive (Histokitt, Glaswarenfabrik Karl Hecht GmbH & Co KG); (**c**) IR spectrum of the adhesive (Histokitt) and paraffin. **Figure S3**. IR spectra of the noncancerous and cancerous (infiltrating ductal carcinoma) human breast tissue. Slices obtained from cryosectioning on the glass window, patient P81. **Table S1**. Assignments of the major bands for Raman and IR spectra of the noncancerous human breast tissue. **Table S2**. Assignments of the major bands for Raman and IR spectra of the cancerous human breast tissue. Click here for file
