# Supplementary material for: Raman imaging at biological interfaces: applications in breast cancer diagnosis
Source: Mol Cancer. 2013 May 24;12:48. doi: 10.1186/1476-4598-12-48 (PMC3681552; doi:10.1186/1476-4598-12-48)
Supplement: Additional file 1: Figure S1 — Illustration of measurement techniques: transmission IR spectroscopy and confocal Raman scattering microscopy. Figure S2. IR spectra of the noncancerous and cancerous (infiltrating ductal carcinoma) human breast tissue slices (patient P81) (a) on the microscope glass of paraffin-embeddeded, nonstained tissue ; (b) the histological samples after deparaffinization, staining and coating with a standard adhesive (Histokitt, Glaswarenfabrik Karl Hecht GmbH & Co KG); (c) IR spectrum of the adhesive (Histokitt) and paraffin. Figure S3. IR spectra of the noncancerous and cancerous (infiltrating ductal carcinoma) human breast tissue. Slices obtained from cryosectioning on the glass window, patient P81. Table S1. Assignments of the major bands for Raman and IR spectra of the noncancerous human breast tissue. Table S2. Assignments of the major bands for Raman and IR spectra of the cancerous human breast tissue. [file 1476-4598-12-48-S1.doc]

**Supplementary materials for publication: *Raman imaging at biological interfaces: Applications in breast cancer diagnosis* by Jakub Surmacki, Jacek Musial, Radzislaw Kordek and Halina Abramczyk.**

**Experimental**

*Patients and samples*

We have studied ductal and lobular carcinoma (in situ and infiltrating) as well as various benign changes including benign dysplastic and neoplastic lesions. The total number of patients was 200. Raman spectroscopy and Raman imaging have been employed to analyze breast cancer specimens. The breast tissue samples were taken during a surgical operation. The research did not affect the course of the operation or treatment of the patients.

In order to visualize and identify tissue structures by Raman imaging and IR spectroscopy we have developed the method of processing surgical specimen. First, we have checked the effect of standard steps in general histology protocols on the Raman measurements, such as formalin fixation, paraffin-embedding, coating to adhere a cover glass to a microscope slide. We have found that using the standard chemical fixative to preserve tissue from degradation – 10% neutral buffered formalin (4% formaldehyde in phosphate buffered saline) does not introduce changes in Raman spectra. This conclusion is based on comparison between the results obtained for fresh tissues (167 patients) and those for formalin - fixed tissues (55 patients). In contrast, the paraffin-embedding is not the appropriate protocol both for Raman and IR measurements. The paraffinization protocol contains a few steps - dehydration with alcohols, clearing with xylene, and infiltrating with the embedding material of paraffin vax, and embedding tissues into paraffin blocks, which may introduce artifacts in Raman spectra resulting from tissue processing. We have checked that the paraffin-embedded samples display the Raman spectra that are similar to those derived from the breast tissue. Clearing washes particular cellular components such as fatty acids out of the tissue, dehydration removes water, both of which are important indicators of pathology and influence or contribute to the onset or progression of specific neoplasias, like breast cancer [1-3]. In order to utilize the paraffin-embedded biological specimen to Raman and IR purposes they must be completely deparaffinized prior to the measurements. However, the standard protocols to ensure complete deparaffinization comprises rehydratation that leads to further washing native fatty acids out of the breast tissue structure and disruption of the hydration profile. We have found that the frozen section procedure, where the frozen fresh tissue (or formalin-fixed tissue) is sliced using a microtome (Microm HM 550, Sermed) into 2-6 μm thick sections, is the most appropriate protocol for Raman and IR measurements. The fresh tissue obtained from the surgery is snapped in liquid nitrogen. Frozen fresh tissue blocks are stored at -80^0^ C until needed for processing. In most cases they are used within a few hours from preparation. The process similar to bread loafing has been employed to cut the surgical specimens into 4 or more sections from the tumour mass and from the safety margins of the excised tumor where no carcinoma was detected by histopathologists. The frozen tissue is cut into thin sections at -25 ºC for unfixed tissue and at -17 ºC for fixed tissue. This procedure ensures that the very thin, adjacent sections represent the same breast structures and the same type of pathology. The thin slices without staining are mounted on a glass slide for Raman measurements, and on BaF_2_ windows for IR measurements. Both for IR and Raman measurements we do not cover the tissue specimen with another layer of glass attached to the sample with a specific adhesive, because it introduces artifacts due to the vibrational spectra of the adhesive in the same spectral range.

The adjacent sections of tissue are mounted on glass slides, stained with hematoxylin and eosin and covered with another layer of glass with a specific adhesive (Histokitt, Glaswarenfabrik Karl Hecht GmbH & Co KG, CAS:1330-20-7) for histology examination. After Raman measurements the slices are stained and examined by pathologists. As the quality of the slides produced by the frozen section is lower for purposes of histology examination than formalin fixed, wax embedded tissue processing - the standard histology processing is additionally performed for a more accurate diagnosis for each patient. The histological analysis was performed by professional medical doctors, board certified as pathologists, from the Medical University of Lodz, Department of Pathology, Chair of Oncology according to the standard histology protocols.

*Raman spectroscopy and imaging*

All Raman images and spectra reported in this study were acquired using a Raman spectrometer Ramanor U1000 (Jobin Yvon, JY) excited with the ion Ar laser (514 nm) and alpha 500 RA (WITec, Ulm, Germany) model consisting of an Olympus microscope, coupled with an UHTS spectrometer and a Newton-CCD camera operating in standard mode with 1024x127 pixels, at -64°C with full vertical binning. The laser beam doubled SHG of the Nd:YAG laser (532 nm) is focused on the sample with a numerical aperture NA of 0.50 to the spot of 200 nm. The average laser excitation power was 10 mW. Before recording the Raman image, the fluorescence in the sample was quenched by illumination with the excitation light at each point. The quenching of the fluorescence was very effective due to the high optical density provided by the light focusing.

The 2D array images of tens of thousands of individual Raman spectra were evaluated by the basis analysis method. In this data analysis method each measured spectrum of the 2D spectral array is compared to basis spectra using a least square fit. Such basis spectra are created from the average spectra from three different areas in the sample. The weight factor in each point is represented as a 2D image of the corresponding color and mixed coloring component.

*Infrared spectroscopy*

IR spectra were recorded using Specord M 80, Germany. The spectra were scanned with the step of 4 cm^-1^.

*Analyzed substances*

Oleic acid, Sigma-Aldrich O1008; linoleic acid, Sigma L2376; α-linolenic acid, Sigma L2376; eicosapentaenoic acid, Sigma E2011; docosahexaenoic acid, Sigma D2534; γ-linolenic acid, Sigma L2378; arachidonic acid, Sigma A3555.

An essential role in histological techniques is played by preservation of tissues, as they naturally occur at the expense of introducing some fixatives, matrices, adhesives, which may generate own vibrational spectra overlapping the signals from the native tissue. To obtain reliable results one must be sure that the method of tissue processing does not distort the vibrational spectra of the studied tissue. Figure S1 illustrates the measurement techniques used in this paper: transmission IR spectroscopy and confocal Raman scattering microscopy.

First, we want to illustrate the effect of various methods of tissue processing on IR and Raman spectra. To check how the preparation of the tissue changes IR and Raman profiles we have compared the spectra for cryosectioned specimens and histological samples after deparaffinization.

Figure S2a shows the typical IR spectra of the noncancerous and cancerous human breast tissue slices on the microscope glass of paraffin-embeddeded, nonstained tissue. Figure S2b shows the same samples after deparaffinization, staining and coating with a standard adhesive Histokitt (Glaswarenfabrik Karl Hecht GmbH & Co KG) used to adhere a cover glass to a microscope slide in order to produce the typical histological sample. Comparison between Figures 2a and 2b shows the results differ markedly and indicate that the method of preparation affects the spectra significantly. Comparison between Figure S2a and S2b with S2c demonstrates that the spectra of the tissues are simply dominated by the paraffin and the adhesive. It indicates that the standard histological samples cannot be used for IR and Raman purposes.

In order to avoid the presented artifacts we use the cryosectioned, and unstained tissue slices placed on BaF_2_ window for transmission IR measurements, which produces no interference in the IR spectra in the studied range (see Figure S2). Both for IR and Raman measurements the tissue specimens are not covered with another layer of glass attached to the sample with a specific adhesive, because it introduces artifact due to the vibrational spectra in the same spectral range as demonstrated in Figure S2. Figure S3 shows the typical IR spectra for the noncancerous and the cancerous breast tissue of the same patient (P81) as in Figure S2, but the tissue is cryosectioned and unstained. In contrast to the results presented in Figure S2, where spectra of the noncancerous and cancerous tissues are almost identical, the IR spectra of the noncancerous and the cancerous breast tissues in Figure S3 differ significantly. To summarize this technical aspect of Raman and IR measurements on the biological tissue ex vivo, we have demonstrated that the frozen sections are recommended for vibrational analysis in contrast to the deparaffinized sections.

**
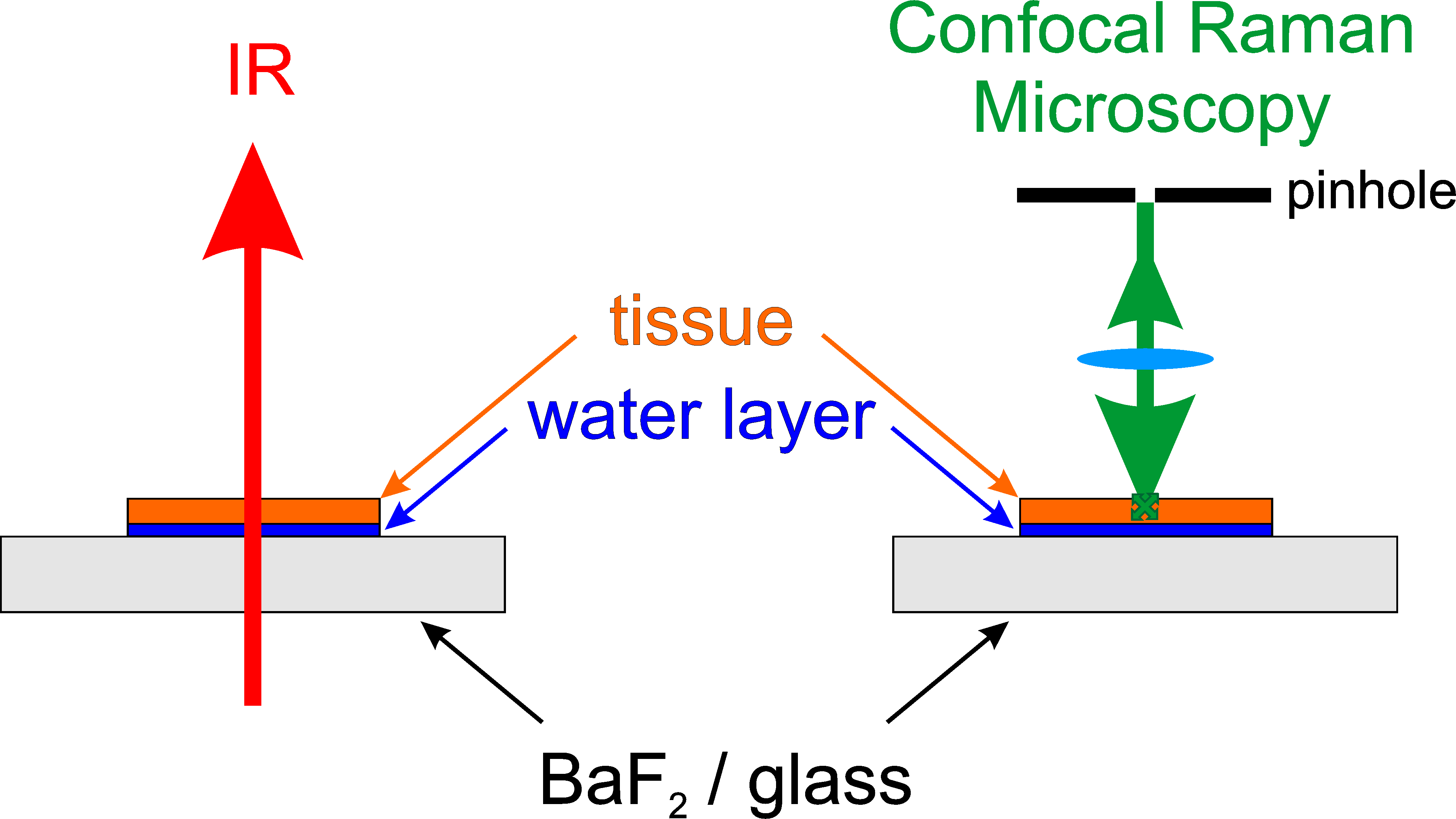
**

**Figure S1.** **Illustration of measurement techniques: transmission IR spectroscopy and confocal Raman scattering microscopy.**


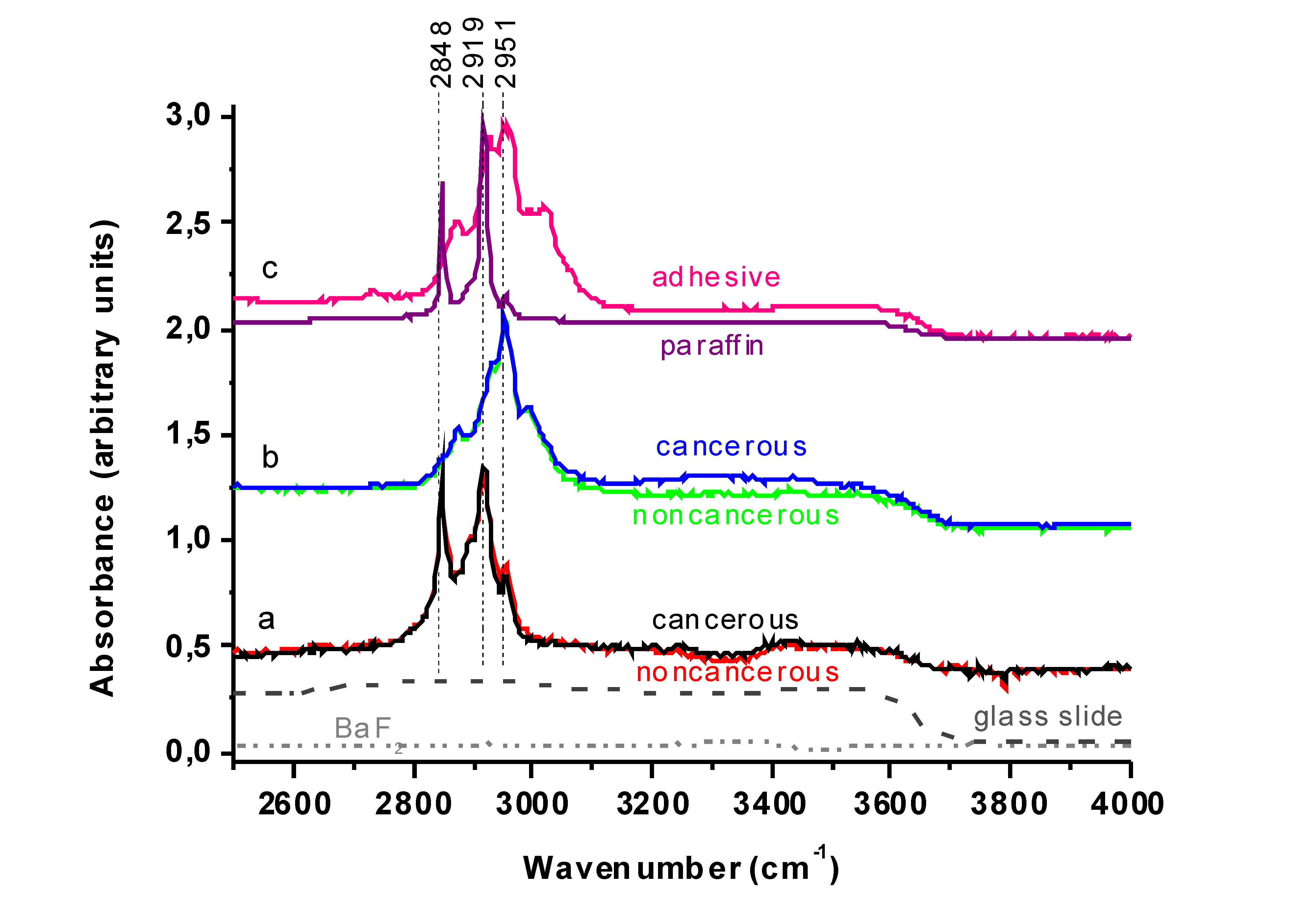


**Figure S2.** **IR spectra of the noncancerous and cancerous (infiltrating ductal carcinoma) human breast tissue slices (patient P81)** (a) on the microscope glass of paraffin-embeddeded, nonstained tissue ; (b) the histological samples after deparaffinization, staining and coating with a standard adhesive (Histokitt, Glaswarenfabrik Karl Hecht GmbH & Co KG); (c) IR spectrum of the adhesive (Histokitt) and paraffin.


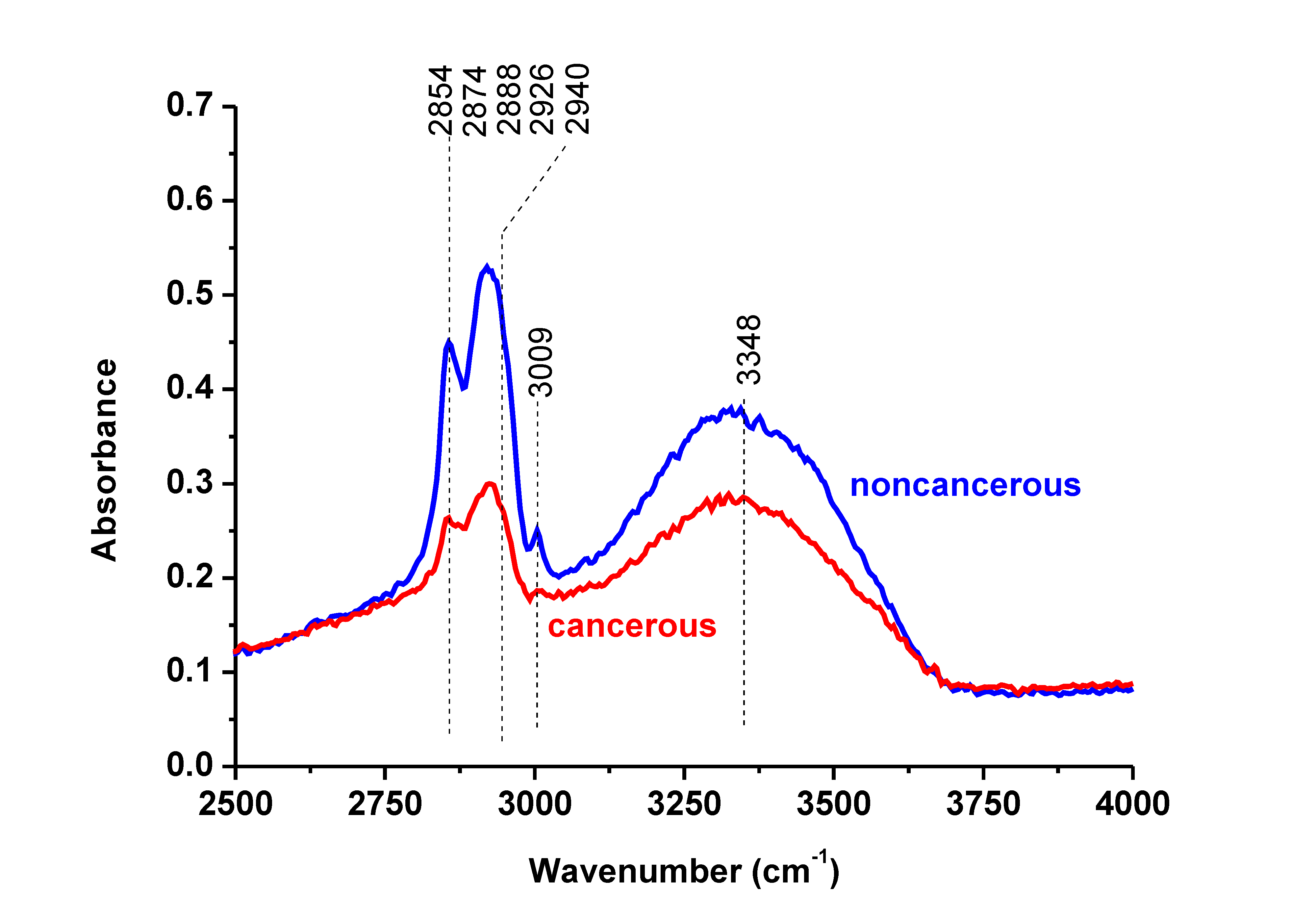


**Figure S3.** **IR spectra of the noncancerous and cancerous (infiltrating ductal carcinoma) human breast tissue**. Slices obtained from cryosectioning on the glass window, patient P81,

**Table 1.** **Assignments of the major bands for Raman and IR spectra of the noncancerous human breast tissue.**

| No | Peak position  Raman [cm^-1^] | Peak position  IR  [cm^-1^] | Major assignments | Mode assignments |
| --- | --- | --- | --- | --- |
| 1 | 727 | 727 | phosphatidylcholine lipids | N^+^(CH_3_)_3_ symmetric stretching [4] |
|  |  |  | lipids | =C-H in plane bending[5] |
|  |  |  | phospholipids | CH2 rocking [6] |
| 2 |  | 842 | tryptophan, protein | Free O-H [6] |
|  |  |  |  | (CCH) aromatic, olefinic [7] |
| 3 | 856 |  | proline, collagen | (C-C) stretching [8] |
| 4 | 877 |  | tryptophan, proteins  phosphatidylcholine, lipids | Tryptophan [4]  antisymmetric stretching [4] |
| 5 | 968 | 972 | lipids | C-C stretching [9]  =C-H out of plane deformation [4] |
|  |  |  | phospholipid | CH=CH bending [6] |
| 6 | 1004 |  | carotenoids | CH_3_ rocking coupled with C-C stretching [10] |
| 7 |  | 1068 | lipids (trans) | C-C stretching [6,9] |
| 8 | 1080 | 1080 |  | C-C stretching [6] |
|  |  |  | phospholipids | stretching (C-C) or stretching (C-O) [10-15] |
| 9 |  | 1098 | phospholipids | PO_2_^-^ symmetric stretching [6,16-25] |
| 10 | 1158 |  | carotenoids | C-C stretching [16-25] |
| 11 |  | 1180 | tyrosine | bending (C-H) [6,10-15] |
| 12 | 1269 | 1240 | phospholipids | _P_^O2-^ antisymmetric stretching [6,16-25],  =C-H in plane deformation [9] |
|  | 1269 |  | amide III, a-helix, collagen, tryptophan | stretching (CN), bending (NH) [8-15] |
| 13 |  | 1280 | lipids | C-H_2_ twisting [4] |
|  |  |  | phospholipids | P=O stretching [6] |
| 14 | 1304 |  |  | C-H_2_ twisting [9] |
|  |  |  | phospholipids | bending (CH_2_) twisting, wagging [13] |
|  |  |  | lipids | H-C= deformation [26] |
| 15 |  | 1344 | collagen | CH_3_, CH_2_ wagging, collage [10-15] |
|  |  |  | nucleic acids | CH_3_, CH_2_ wagging [10-15] |
| 16 |  | 1370 | amide S | CαC-H bending mixed with C-N stretch and N-H in plane bending [4] |
| 17 | 1444 | 1444 | lipids | CH_2_ scissoring  CH_2_ deformation [9,16-25]  CH_2_  bending [4] |
|  | 1444 | 1444 | phospholipids | δ (CH_2_) scissoring [10-15] |
|  | 1444 | 1444 | collagen | δ (CH_2_), δ (CH_3_) [10-15] |
| 18 |  | 1465 |  |  |
| 19 | 1518 |  | carotenoids | C=C stretching [16-25] |
| 20 |  | 1544 | tryptophan, proteins | Tryptophan [27]  N-H bending (amide II), C-N stretching [28] |
| 21 | 1600-1700 |  | amide I, α-helix conformation | C=O  C-N stretching [16-25] |
| 22 |  | 1642 | water | OH bending [6,16-25] |
| 23 | 1660 | 1653 | unsaturated bonds of lipids | C=C stretching [9]  C=C stretching (1660 for *cis*, 1654 for *trans*) [4] |
|  |  |  | unsaturated lipids | C=C band [9]; antisymmetric stretching (C=C) *cis*, lipids [6]; antisymmetric stretching (C=C), unsaturated bonds of lipids [16-25] |
| 24 | 1750 | 1739 | lipids | C=O stretching [16-25] |
| 25 | 2854 | 2854 | saturated bonds of lipids, fatty acids | C-H stretching,  CH_2_ symmetric stretching [6,7,9] |
| 26 |  | 2874 | unsaturated bonds of lipids, fatty acids  lipids | C-H stretching, C-H_2_ antisymmetric [9]  CH_3_ symmetric stretching [29] |
| 27 | 2888 | 2888 | saturated bonds of lipids, fatty acids | (CH_2_)C-H antisymmetric stretching [16-25] |
|  |  |  | lipids | stretching (CH_2_) antisymmetric [7] |
| 28 | 2902 | 2902 |  | C-H stretching,C-H_3_ symmetric [9] |
| 29 | 2926 | 2926 | saturated bonds of lipids, fatty acids and polipeptide  proteins | CH_2_  antisymmetric stretching [6,16-25]  C-H stretching, C-H_3_ symmetric stretching [4,9,29] |
| 30 | 2940 | 2936 | aromatic and aliphatic amino acids, charged amino acids, proline, threonine, histidine, lipids, proteins | C-H stretching [27] |
| 31 |  | 2960 |  | C-H stretching,C-H_3_ antisymmetric [6,9]  out of plane chain and antisymmetric  C-H_3_ stretching [4,29] |
| 32 | 3009 |  | unsaturated bonds of lipids, fatty acids | H -C=C stretching [9,29] |
| 33 |  | 3348 | water | O-H stretching [16-25] |

Table 2. Assignments of the major bands for Raman and IR spectra of the cancerous human breast tissue.

| No | Peak position  Raman [cm^-1^] | Peak position  IR  [cm^-1^] | Major assignments | Mode assignments |
| --- | --- | --- | --- | --- |
| 1 | 558 |  | tryptophan, protein | tryptophan [6] |
| 2 |  | 727 | phosphatidylcholine lipids | N^+^(CH_3_)_3_ symmetric stretching [4] |
|  |  | 727 | lipid | =C-H in plane bend [5] |
|  |  | 727 | phospholipids | CH_2_ rocking [6] |
| 3 |  | 850 | tryptophan, protein | Free O-H [6] |
|  |  |  |  | deformation (CCH) aromatic, olefinic [7] |
| 4 | 856 |  | proline, collagen | (C-C) stretching [8] |
| 5 | 877 | 877 | tryptophan, protein  phosphatidylcholine lipids | tryptophan [4]  antisymmetric stretching [4] |
| 6 | 944 | 952 | lipids | C-C stretching [9]  =C-H out of plane deformation [9] |
|  |  | 952 | phospholipid | CH=CH bending [6] |
| 7 |  | 1068 | lipids (trans) | C-C stretching [6,9] |
| 8 |  | 1080 |  | C-C stretching [9] |
|  |  |  | phospholipids | stretch (C-C) or stretch (C-O) [10-15] |
| 9 | 1098 | 1098 | phospholipids | symmetric stretching [6,16-25] |
| 10 |  | 1180 | tyrosine | bending (C-H) [16-25] |
| 11 | 1259 | 1240 | phospholipids | _P_^O2-^ antisymmetric stretch [6,16-25]  =C-H in plane deformation [9] |
|  | 1269 |  | amide III, a-helix, collagen, tryptophan | stretch (CN), bending (NH) [8-15] |
| 12 |  | 1280 | lipids | C-H_2_ twisting [4] |
|  |  | 1280 | phospholipids | P=O stretching [6] |
| 13 | 1304 |  |  | C-H_2_ twisting [9] |
|  | 1304 |  | phospholipids | bending (CH_2_) twisting, wagging [6] |
|  | 1304 |  | lipids | H-C= deformation [26] |
| 14 | 1334 | 1344 | collagen | CH_3_CH_2_ wagging, collage [10-15] |
|  |  | 1344 | nucleic acids | CH_3_CH_2_ wagging [10-15] |
| 16 | 1444 | 1444 | lipids | CH_2_ scissoring  CH_2_ deformation [9,16-25]  CH_2_  bending [4] |
|  | 1444 | 1444 | phospholipids | δ (CH_2_) scissoring [10-15] |
|  | 1444 | 1444 | collagen | δ (CH_2_), δ (CH_3_) [10-15] |
| 17 |  | 1544 | tryptophan, proteins | Tryptophan [27]  N-H bending (amide II), C-N stretching [28] |
|  |  | 1544 | Amide II, polypeptide | NH bending in plane, CN stretching [6] |
| 18 | 1600-1700 |  | amide I, α-helix conformation | C=O  C-N stretching [16-25] |
| 19 |  | 1642 | water | OH bending [6,16-25] |
| 20 | 1660 | 1652 | unsaturated bonds of lipids | C=C stretching [9]  C=C stretching (1660 for *cis*, 1654 for *trans*) [4] |
|  |  |  |  | antysymetric stretching (C=C), unsaturated bonds of lipids [6, 16-25] |
| 21 |  | 1740 | lipids | C=O stretching [9,16-25] |
| 22 | 2854 | 2854 | saturated bonds of lipids,fatty acids | C-H stretching,  C-H_2_ symmetric [6,7,9] |
| 23 | 2874 | 2874 | unsaturated bonds of lipids, fatty acids  lipids | C-H stretching, C-H_2_ antisymmetric [9]  CH_3_ symmetric stretching [29] |
| 24 | 2888 | 2888 | saturated bonds of lipids, fatty acids | (CH_2_)C-H antisymmetric stretching [16-25] |
|  |  |  | lipids | (CH_2_) antisymmetric stretching [7] |
| 25 | 2902 | 2902 |  | C-H stretching,C-H_3_ sym [9] |
| 26 | 2926 | 2926 | saturated bonds of lipids, fatty acids and polipeptide  proteins | CH_2_  antisymmetric stretching [6,16-25]  C-H stretching,C-H_3_ sym [9]  C-H_3_ symmetric stretching [4,29] |
| 27 | 2940 | 2936 | aromatic and aliphatic amino acids, charged amino acids, proline, threonine, histidine, lipids, proteins | C-H stretching [27] |
| 28 |  | 2960 |  | C-H stretching,C-H_3_ asym [6]  out of plane chain and antisymmetric  C-H_3_ stretching [4,29] |
| 29 | 3009 | 3008 | unsaturated bonds of lipids, fatty acids | H -C=C stretching [9,29] |
| 30 | 3056 | 3056 | proteins | amide II [6] |
|  |  |  |  | stretching (CH), olefinic [7,30] |
| 31 | 3232 |  | DNA, proteins | N-H stretching [29] |
| 32 | 3311 | 3348 | water | O-H stretching [16-25] |

**References**

1. Abramczyk H, Brozek-Pluska B, Surmacki J, Jablonska-Gajewicz J, Kordek R: **Hydrogen bonds of interfacial water in human breast cancer tissue compared to lipid and DNA interfaces.** *JBPC* 2011, **2**:158-169.
2. Brozek-Pluska B, Jablonska-Gajewicz J, Kordek R, Abramczyk H: **Phase transitions in oleic acid and in human breast tissue as studied by Raman spectroscopy and Raman imaging.** *J Med Chem* 2011, **54:**3386-3392.
3. Menendez JA, Ropero S, Lupu R, Colomer R: **Dietary fatty acids regulate the activation status of Her-2/neu (c-erbB-2) oncogene in breast cancer cells.** *Ann Oncol* 2004, **15**:1719-1721.
4. Petersen FNR, Nielsen CH: **Raman spectroscopy as a tool for investigating lipid-protein interactions.** *Spectroscopy.* 2009, **24**:1-8.
5. Kneipp J, Tom BS, Kliffen M, Marian MP, Puppels G. **Characterization of breast duct epithelia: a Raman spectroscopic study.** *Vib Spectrosc* 2003, **32**:67-74.
6. Parker FS: *Applications of Infrared Raman, and Resonance Raman Spectroscopy in Biochemistry.* Plenum Press: New York;1983, p. 421-480.
7. Edwards HGM, Williams AC, Barry BW: **Potential applications of FT-Raman spectroscopy for dermatological diagnostics.** *J Mol Struc* 1995, **347**:379-387.
8. Frank CJ, McCreery RL, Redd DC: **Raman spectroscopy of normal and diseased human breast tissues.** *Anal Chem* 1995, **67**:777-783.
9. Frank CJ, Redd DCB, Gansler TS, McCreery RL: **Characterization of human breast biopsy specimens with near-IR Raman spectroscopy.** *Anal Chem* 1994, **66**:319-26.
10. Mahadevan-Jansen A, Mitchell MF, Ramanujam N, Malpica A, Thomsen S, Utzinger U, Richards-Kortum R: **Near-infrared Raman spectroscopy for *in vitro* detection of cervical precancers.** *Photochem Photobiol* 1998, **68**:123-132.
11. Mizuno A, Kitajima H, Kawauchi K, Muraishi S, Ozaki Y: **Nearinfrared Fourier transform Raman spectroscopic study of human brain tissues and tumors.** *J Raman Spectrosc* 1994, **25**:25-29.
12. Stone N, Stavroulaki P, Kendall C, Birchall M, Barr H: **Raman spectroscopy for early detection of laryngeal malignancy: preliminary results.** *Laryngoscope* 2000, **110**:1756-1763.
13. Shim MG, Wong LKS, Marcon NE, Wilson BC: **The effects of ex vivo handling procedures on the near-infrared Raman spectra of normal mammalian tissues.** *Photochem Photobiol* 1996, **63**:662-671.
14. Keller S, Schrader B, Hoffmann A, Schrader W, Metz K, Rehlaender A, Pahnke J, Ruwe M, Budach W: **Application of near-infrared Fourier transform Raman spectroscopy in medical research.** *J Raman Spectrosc* 1994, **25**:663-671.
15. Dollish FR, Fateley WG, Bentley FF: Characteristic Raman frequencies of organic compounds. New York, NY: Wiley-Interscience;1974, p. 215-283.
16. Abramczyk H, Brozek-Pluska B, Surmacki J, Jablonska J, Kordek R: **The label-free Raman imaging of human breast cancer.** *J Mol Liq* 2011, **164**:123-131.
17. Lasch P, Naumann D: **Spatial resolution in infrared microspectroscopic imaging of tissues.** *Biochim Biophys Acta* 2006, **1758**:814-829.
18. Kretlow A, Wang Q, Kneipp J, Lasch P, Beekes M, Miller L, Naumann D: **FTIR-microspectroscopy of prion-infected nervous tissue.** *Biochim Biophys Acta* 2006, **1758**:948-959.
19. Fabian H, Thi NAN, Eiden M, Lasch P, Schmitt J, Naumann D: **Diagnosing benign and cancerous lesions in breast tissue sections by using IR microspectroscopy.** *Biochim Biophys Acta* 2006, **1758**:874-882.
20. Lasch P, Haensch W, Naumann D, Diem M: **Imaging of colorectal adenocarcinoma using FT_IR microspectroscopy and cluster analysis.** *Biochim Biophys Acta* 2004, **1688**:176-186.
21. Lasch P, Pacifico A, Diem M: **Spatially resolved IR microspectroscopy of single cells.** *Biopolymers* 2002, **67**:335-338.
22. Miller LM, Dumas P: **Chemical imaging of biological tissue with synchrotron infrared light.** *Biochim Biophys Acta* 2006, **1758**:846-857.
23. Miller LM, Dumas P, Jamin N, Teillaud JL, Bantignies JL, Carr GL: **Applications of synchrotron infrared microspectroscopy to the study of biological cells and tissues.** *Inst Phys Conf Ser Sponsored by Micro Beam Anal* 2000, **165**:75-76.
24. Gazi E, Gardner P, Lockyer NP, Hart CA, Clarke NW, Brown MD: **Probing lipid Translocation between Adipocytes and prostate cancer cells with imaging FTIR microspectroscopy.** *J Lipid Res* 2007, **48**:1846-1856.
25. Mahadevan-Jansen A, Richards-Kortum R: **Raman spectroscopy for cancer detection: a review.** *Proc. IEEE/EMBS* 1997, 2722-2728.
26. Verier S, Nothinger I, Polak, JM, Hench LL: **In situ monitoring of cell death using Raman microscopy.** *Biopolymers* 2004, **74**:157-162.
27. Howell NK, Arteaga GE, Nakai S, Li-Chan ECY: **Raman spectral analysis in the C-H stretching region of proteins and amino acids for investigation of hydrophobic interactions.** *J Agric Food Chem* 1999, **47**:924-933.
28. El-Bahy GMS: **FTIR and Raman spectroscopy study of fenugreek (Trigonella foenum graecum L.) seeds.** *J* Appl Spectrosc 2005, **72**:111-116.
29. García-Flores AF, Raniero L, Canevari RA, Jalkanen KJ, Bitar RA, Martinho HS, Martin AA: **High wavenumber FT-Raman spectroscopy for in vivo and ex-vivo measurements of breast cancer.** *Theor Chem Acc* 2011, DOI:10.1007/s00214-011-0925-9.
30. Camerlingo C, Zenone F, Perna G, Capozzi V, Cirillo N, Gaeta GM, Lepore M: **An investigation on micro-Raman spectra and wavelet data analysis for pemphigus vulgaris follow-up monitoring.** *Sensors* 2008, **8**:3656-3364.
